# Determining the Effect of Different Reproduction Factors on the Yield and Hatching of Tenebrio Molitor Eggs

**DOI:** 10.3390/insects13070615

**Published:** 2022-07-08

**Authors:** Lotte Frooninckx, Siebe Berrens, Meggie Van Peer, Ann Wuyts, Laurens Broeckx, Sabine Van Miert

**Affiliations:** RADIUS, Thomas More University of Applied Sciences, Kleinhoefstraat 4, 2440 Geel, Belgium; siebe.berrens@thomasmore.be (S.B.); meggie.vanpeer@thomasmore.be (M.V.P.); ann.wuyts@thomasmore.be (A.W.); laurens.broeckx@thomasmore.be (L.B.); sabine.vanmiert@thomasmore.be (S.V.M.)

**Keywords:** *Tenebrio molitor*, insect farming, insect breeding, reproduction, yellow mealworm, oviposition

## Abstract

**Simple Summary:**

This study investigated the effect of various factors on the reproduction of the yellow mealworm *Tenebrio molitor*. More specifically, the effect of (1) the use of a grid, (2) oviposition duration, (3) age of the beetles and (4) beetle density were examined. For these parameters, it was investigated how they influence the number of eggs laid per beetle as well as the egg hatching rate and time. All these factors had a clear impact on the number of eggs. The results of this study will help producers to optimize their breeding method in order to obtain higher yields.

**Abstract:**

Whereas the production of conventional livestock can rely on years of knowledge and experience, the mealworm production industry is still in its early stages. Although the yellow mealworm *Tenebrio molitor* has been cultivated as feed for pets and zoo animals for quite some time, the optimization of the reproduction process has not been widely explored. For reproduction, beetles are placed in crates supplemented with a substrate to oviposit their eggs. After a specified time, the beetles are removed, and the eggs are further cultivated to develop into larvae. Factors such as oviposition duration and beetle density influence the number of produced mealworms per beetle, partly due to the cannibalistic behavior of the beetles. However, reproductive success has mostly been assessed by determining the number of offspring several weeks after oviposition. As a result, the number of eggs laid and their hatch rates are unknown. In this research, eggs are separated from the oviposition substrate, i.e., harvested. This approach allows to determine the influence of beetle density, oviposition duration, beetle age and the use of a grid during oviposition on the number of eggs produced and the egg hatching rate and timing thereof. In addition, the influence of the harvesting method on the hatching of *T. molitor*′s eggs was determined.

## 1. Introduction

The global human population is growing and is expected to reach 9.7 billion by 2050 [1]. Along with this growth, the demand for natural resources is rising. This increase puts pressure on our ecosystem and is threatening the food supply [2]. In light of this problem, efforts are being made to make current food production processes more sustainable or to find sustainable alternatives. With regard to the production of food and feed, insects are considered a promising alternative [2,3,4]. Insects are capable of converting low-value raw materials into sustainable biomass that can be used in food, feed, or biochemicals [5,6]. Since van Huis et al. drew attention to the potential of insects in food and feed, several companies have engaged in large-scale insect production around the world [4]. The yellow mealworm *Tenebrio molitor* (Coleoptera: Tenebrionidae) is one of the most promising insect species for industrial production [7]. The yellow mealworm has generally been considered as a pest because they often infest and damage stored grains and grain products [8]. Nevertheless, the larvae have been reared for some time in small-scale facilities as feed for birds, reptiles, and a variety of small animals. However, since this sector is still in its early stages, mealworm producers have to invest in automation and optimization of the production process in order to be competitive with conventional feed and food products [7].

Continuous production of fertile eggs is one of the essential factors for sustainable mealworm production. During their life cycle, mealworms undergo a complete metamorphosis that consists of four different life stages: (1) egg, (2) larva, (3) pupa and (4) adult beetle [9]. The amount of time a mealworm spends in each stage can vary greatly due to environmental factors such as temperature, humidity, food, and access to water [10]. When kept at 28 °C and 60% relative humidity, female beetles become fertile 2–4 days after emergence [11]. Mating stimulates oocyte production, so yellow mealworm females that mate multiple times are more fertile [12]. In virgin females, vitellogenesis stops approximately one week after emergence, whereas mated females continue to produce oocytes [11]. Mated female beetles are continuously receptive throughout their adult lives [12]. However, the peak of reproductive potential is reached in the second week after emergence. After three weeks, on average, the egg production drops sharply [13]. On average, female beetles produce a total of 250 to 500 eggs per individual, which they oviposit singly or in small clusters [14]. Cotton [9] found that beetles lay up to 12 eggs a day or 576 eggs throughout their life.

The yield of juvenile mealworms depends on different factors during reproduction, including the oviposition substrate. In their natural environment, *T. molitor* beetles deposit their eggs buried in a food source [11]. Rumbos et al. [15] tested forty-four commodities as potential oviposition and feeding substrates for *T. molitor*. The highest amounts of larvae were produced on wheat bran, milk-based feed, egg-layer hen feed, buckwheat flour and rye flour. Moreover, similar findings were reported for larval development as these substrates also showed the best growth rates [15]. These results confirm the preference of oviposition in substrates that allow feeding of the offspring immediately after hatching. This principle is used in mealworm reproduction facilities. The beetles are provided with an oviposition substrate that is also used as a feed source. After oviposition, beetles are removed from the rearing container and offspring are cultivated for further development. However, as the eggshell contains a sticky material, the eggs are not only attached to the feed source but often also to the walls and floors of the rearing crate [11,14], making it almost impossible to harvest the eggs. Therefore, the offspring are cultivated in the same substrate and rearing container as used for oviposition.

Using this method might be relatively simple; however, it is not optimal. First of all, since the attached eggs cannot be harvested without damaging them, the exact yield is undetermined, leading to inefficient feeding and variable production quantities per crate. Secondly, *T. molitor* beetles are cannibalistic, as occurs in all Tenebrionidae. *T. molitor* beetles eat their eggs, leading to decreased egg yield when applying this technique as the beetles are able to damage the eggs during the oviposition period, especially at high beetle densities [13,16,17,18]. For example, both Morales-Ramos et al. [13] and Berggreen et al. [18] observed a decrease in reproduction with increasing beetle density, and Deruytter et al. [19] observed a reduction in the amount of mealworm produced per female with increasing beetle density. Not only the beetle density but also the duration of oviposition seems to have a large influence on the amount of produced mealworms. Increasing the oviposition time led to a decrease in produced larvae/female beetle in both the study of Berggreen et al. [18] and Deruytter et al. [19]. Concretely, oviposition duration and beetle density appreciably affect reproduction.

Reproductive success can be optimized by using optimal beetle densities and optimal duration of oviposition. In the study of Morales-Ramos et al. [13], an optimal beetle density of 0.14 beetles/cm² was found. However, Berggreen et al. [18] and Deruytter et al. [19] could not find optimal beetle densities. Although increasing the oviposition time leads to a decrease in produced larvae/female beetle per day, the total yield per container still increases in the abovementioned studies. However, both studies suggested allowing oviposition by the beetles for no longer than 4 days as this leads to large variabilities in the age of the produced mealworms [18,19]. In addition, using a grid during reproduction might have a positive impact on the yield as this technique prevents egg cannibalism by separating the beetles from the laid eggs [19].

In most of the studies investigating *T. molitor* reproduction, the success of the beetles was assessed by determining the number of mealworms a few weeks after the beetles were allowed to oviposit, as the mealworms are then big enough to be handled. However, this method fails to assess the number of eggs laid, the hatching rate and timing thereof. Therefore, the objective of this study is to determine the influence of different reproduction factors on the egg-yield and hatching rate of *T. molitor*′s eggs. Different reproduction factors investigated in this study include (1) the use of a grid during oviposition, (2) oviposition duration, (3) beetle age and (4) beetle density. In addition, we investigated the effect of different harvesting methods on *T. molitor*′s egg hatching rate and timing thereof.

## 2. Materials and Methods

### 2.1. Insects Origin and Maintenance

Ten-week-old *T. molitor* larvae were obtained from De Smedt Insects (Tessenderlo, Belgium). Larvae were maintained in plastic crates (l 60 × w 40 × h 33 cm) (Engels Logistics, Beringen, Belgium) with a rectangular opening (30 × 15 cm) at the front for ventilation. Upon pupation, pupae were collected daily by separating them from the larvae using a FIAP Profibreed grid with a gap width of 3.5 mm (Ursensollen, Germany). Beetles were separated from the pupae by adding egg cartons to the pupae crates. Beetles attached to the egg cartons were collected daily by tapping them off into beetle collection crates. All *T. molitor* stages were maintained in a climate room with a mean temperature of 26 °C and 60% relative humidity. It was always dark in the climate chamber, except during feeding.

### 2.2. Experimental Design

**Figure 1:**
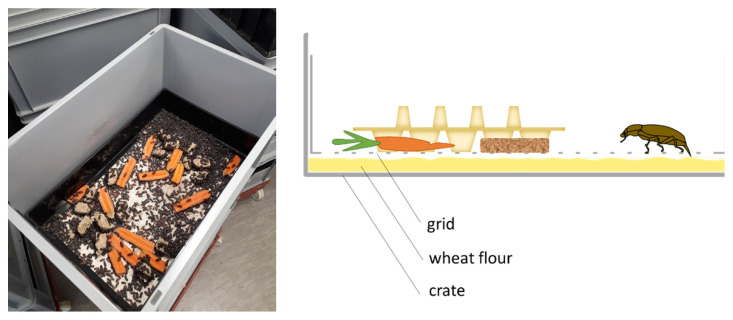
Photo and graphic representation of oviposition grid.

Two experiments were conducted in order to study and distinguish the effect of different reproduction factors on the oviposition and hatching of *T. molitor*′s eggs (Table 1). In the first experiment, the influence of the use of an oviposition grid (Figure 1) was investigated as well as oviposition duration and beetle age. In the second experiment, the same was investigated, with the addition of different beetle densities.

The first experiment was performed by placing 200 g of beetles in each crate (l 60 × w 40 × h 33 cm) (Engels Logistics, Beringen, Belgium) with a rectangular opening (30 × 15 cm) at the front to allow ventilation. The inner surface of the crates was 1997.64 cm^2^, resulting in a beetle density of approximately 0.1 g beetles/cm² (equal to 0.788 beetles/cm²). In total, 6 batches of beetles were setup: 3 batches with an oviposition grid and 3 batches without an oviposition grid. Each beetle batch was allowed to lay eggs for 2, 4 and 8 days. After each oviposition period (2, 4 or 8 days), beetles were removed from the crates and transferred to a new crate with a different oviposition duration. At each transfer, dead beetles were replaced with new beetles from the same age. By implementing a rotation scheme for the different oviposition durations among the beetle batches, the effect of beetle age was included in the experimental setup (Table 1). This setup resulted in 18 different treatments.

For the second experiment, beetles were placed in crates at three densities, i.e., 0.025 g/cm² (equal to 0.197 beetles/cm²), 0.1 g beetles/cm² (equal to 0.788 beetles/cm^2^) and 0.2 g beetles/cm² (equal to 1.576 beetles/cm²). For each density, 6 beetle batches were set up: 3 batches with an oviposition grid and 3 batches without an oviposition grid. Per density, beetles were allowed to lay eggs for 2, 4, or 8 days. After each oviposition period (2, 4 or 8 days), beetles were removed from the crates and transferred to a new crate with a different oviposition duration. At each transfer, dead beetles were replaced with new beetles from the same age. By implementing a rotation scheme for the different oviposition durations among the beetle batches, the effect of beetle age was included in the experimental setup (Table 1). This setup resulted in 54 different treatments.

Beetles were between 1 and 2 weeks old at the start of the experiment. Beetle crates were filled with 1.5 kg of wheat flour as an oviposition substrate. For the treatments with an oviposition grid, the grid was placed directly on top of the wheat flour. This grid consisted of a stainless steel mesh (mesh size 2 mm), which was mounted in a plastic frame that could be inserted into the beetle crates (Figure 1). Egg cartons were placed upon the wheat flour or the grids to increase the surface and to prevent clumping of the wheat flour when providing the moisture source. Carrots and Weetabix (Weetabix Limited, Burton Latimer, UK) were provided as feed ad libitum and were replaced every 2 days.

### 2.3. Harvesting of The Eggs

Eggs were collected from the wheat flour using a 500 µm sieve with a diameter of 30 cm (Prosep, Zaventem, Belgium). The sieve was mounted on a HAVER Sieve Shaker EML 315 digital plus T for dry sieving (Oelde, Germany).

The number of eggs present after sieving was quantified by weight. Due to the sieving process, small insect and feed remains were also collected during the harvesting of the eggs. To correct for this, the weight of the harvested eggs, as well as the number of eggs present therein, had to be determined for every treatment. Therefore, three homogeneous samples of the harvest (approximately 0.1 g each) were taken from which the total weight and number of eggs were determined. Standard deviation between subsamples was less than 10% of the mean.

In order to investigate the effect on the hatching rate of the above ′automated′ harvesting method using a vibrating screen, eggs were also collected manually directly from the crates using a paintbrush.

### 2.4. Determining Egg Hatching 

In order to determine the hatching rates of the harvested eggs, 24 eggs were individually isolated in the wells of a 96 well-plate (Greiner Bio-One, Austria, Kremsmünster) per treatment. Eggs were incubated in a climate chamber with a mean temperature of 26 °C and 60% relative humidity. Egg hatching was inspected daily for 14 days. Time of hatching is expressed in days, with the day of harvest as the starting point. 

Hatching rate was calculated as:

(1) $$hatching\;\left(\%\right)=\frac{number\;of\;hatched\;eggs}{number\;of\;eggs}\times 100\;\%$$

### 2.5. Data Analysis

All statistics were computed using SAS JMP 16.0.0 statistical software (SAS Institute Inc., Cary, NC, USA). Two experiments were conducted, which were analyzed separately. 

Independent variables investigated were harvesting technique (categorical), presence of grid (categorical), oviposition duration (continuous), beetle density (continuous) and beetle age (continuous).

Dependent variables were the number of eggs (expressed as the number of eggs per beetle per day), hatching rate and timing thereof. 

For experiment 1, the influence of the harvesting technique and presence of grid on hatching rate was investigated with a two-way ANOVA. Data were tested for normality using a Shapiro–Wilk′s test and Levene′s test to examine the homogeneity of variance. To determine which means were different, a two-sample *t*-test was used for post hoc comparison.

For experiment 1, the effect of the presence of grid, oviposition duration and beetle age on the number of eggs per beetle per day, hatching rate and timing thereof was investigated. In experiment 2, the influence of density was included. For both experiments, a stepwise multiple linear regression was performed to investigate the influence of the independent parameters and the interaction thereof. Before performing the multiple linear regression, each independent variable was examined individually against the dependent variables to see if a correlation could be observed (Supplementary Materials). This showed that mean beetle age showed an exponential correlation with the number of eggs per beetle per day. Therefore, this parameter was transformed (ln(x − 10.5)) for the multiple linear regression. Non-significant effects (*p* < 0.05) were excluded from the model.

## 3. Results

### 3.1. Effect of Harvesting Method on Hatching

**Figure 2:**
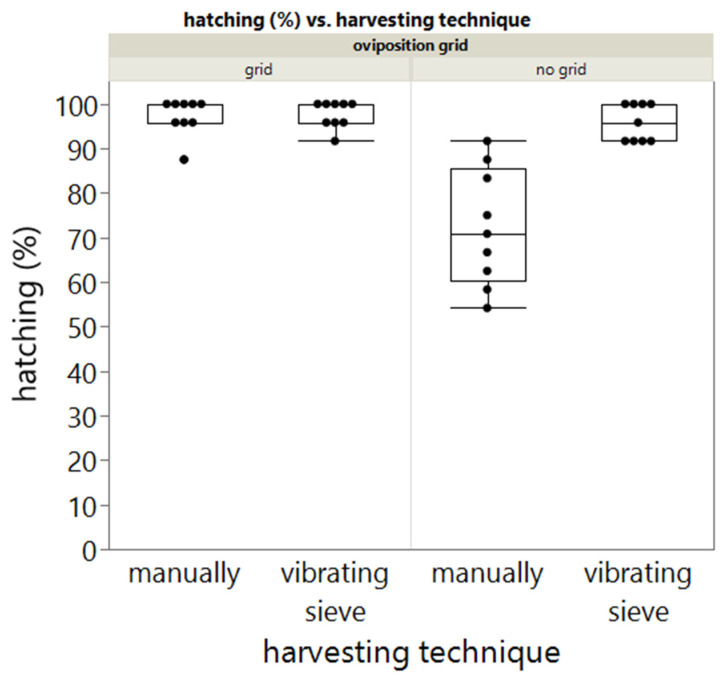
Boxplot representing the effect of harvesting method and presence of grid on egg hatching. Points represent individual measurements.

In order to check if harvesting the eggs with the vibrating sieve would damage the eggs and therefore lower the hatching rate, the hatching of eggs that were manually sampled from the egg substrate and harvested with the vibrating 500 µm sieve were monitored in experiment 1 (Figure 2). 

A two-way ANOVA revealed that there was a statistically significant interaction between the effects of harvesting technique and use of an oviposition grid (F(3, 32) = 25.33, *p* < 0.0001). Both harvesting technique (*p* < 0.0001) and use of an oviposition grid (*p* < 0.0001) have a significant effect on the hatching rate. A significant interaction is observed between the two parameters (*p* < 0.001). When a grid is present, no significant difference (*t*-test, t(14.6) = 0.26988, *p* = 0.7910) is present between manual harvesting (mean = 97.221, SD = 4.167, *n* = 9) and harvesting using a vibrating sieve (mean = 97.684, SD = 3.027, *n* = 9). When no grid is present, a significant difference (*t*-test, t(9.6)= 5.12612, *p* < 0.0005) is present between manual harvesting (mean = 72.222, SD = 13.176, *n* = 9) and harvesting using a vibrating sieve (mean = 95.834, SD = 4.165, *n* = 9). 

### 3.2. Experiment 1: Influence of Presence of Grid, Oviposition Duration and Beetle Age on Number of Eggs per Beetle per Day, Hatching Rate and Timing Thereof

For experiment 1, presence of grid, oviposition duration and beetle age were varied in the experimental set up (Table 1). Influence of these variables on the number of eggs per beetle per day, hatching rate and timing thereof was investigated using a multiple stepwise linear regression model. These variables statistically significantly predicted number of eggs per beetle per day (F(3,14) = 34.9947, *p* < 0.0001, R^2^ = 0.88). 

For the number of eggs per beetle per day without a grid, the estimated regression equation is:

(2) $$\begin{array}{c} number\;of\;eggs\;per\;beetle\;per\;day=\\ 7.297\\ –0.19\times \;oviposition\;duration\;\left(days\right)\\ –\;1.36\times ln\left(mean\;age\;of\;beetles\;\left(days\right)-10.5\right) \end{array}$$

When an oviposition grid is used, the estimated regression equation is:

(3) $$\begin{array}{c} number\;of\;eggs\;per\;beetle\;per\;day=\\ 9.471\\ –\;0.19\times oviposition\;duration\;\left(days\right)\\ –\;1.36\times ln\left(mean\;age\;of\;beetles\;\left(days\right)-10.5\right) \end{array}$$

Number of eggs per beetle per day significantly correlate to the presence of grid, oviposition duration and beetle age (Table 2).

**Figure 3:**
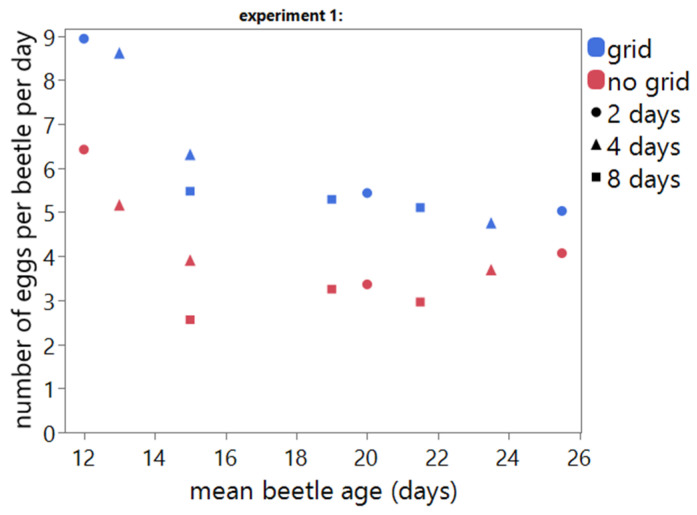
Scatterplot of the number of eggs per beetle per day vs. mean beetle age (days) for experiment 1. Points represent individual measurements. The use of an oviposition grid is indicated in blue (grid) and red (no grid). Oviposition duration is indicated with symbols ● = 2 days, ▲ = 4 days and ■ = 8 days.

The presence of a grid has a positive influence on the number of eggs per beetle per day for all tested oviposition durations and beetle ages (intercept increases from 7.297 (8.384 − 1.087) to 9.471 (8.384 + 1.087) when a grid is added to the crates) (Figure 3). Oviposition duration and beetle age have a negative influence on the number of eggs per beetle per day.

For hatching only, beetle age had a significant linear correlation (F(1,16) = 7.1448, *p* = 0.0167, R^2^ = 0.31). Increasing beetle age has a small positive influence (slope 0.004) on the hatching rate (Table 3). 

For mean hatching time, only oviposition duration had a significant linear correlation (F(1,16) = 54.7762, *p* < 0.0001, R^2^ = 0.77). Increasing oviposition duration has a negative influence (slope −0.318223) on the mean hatching time (Table 4). 

### 3.3. Experiment 2: Influence of Presence of Grid, Oviposition Duration, Beetle Age and Beetle Density on Number of Eggs per Beetle per Day, Hatching Rate and Timing Thereof

For experiment 2, presence of grid, oviposition duration, beetle age and beetle density on the number of eggs per beetle per day, hatching rate and timing thereof were investigated using a multiple stepwise linear regression model. 

For the number of eggs per beetle per day without a grid, the estimated regression equation is displayed below:

(4) $$\begin{array}{c} number\;of\;eggs\;per\;beetle\;per\;day=\\ 9.306\\ -0.882\times beetle\;density\;\left(number/cm²\right)\\ -0.162\times oviposition\;duration\;\left(days\right)\\ -2\times \ln\left(mean\;age\;of\;beetles\;\left(days\right)-10.5\right)\;\\ +0.695\;x\;beetle\;density\;\left(number/cm^{2}\right)\;\\ \times \left(\ln\left(mean\;age\;of\;beetles\;\left(days\right)-10.5\right)-1.848\right)\\ +0.817\;x\;\left(ln\left(mean\;age\;of\;beetles\;\left(days\right)-10.5\right)-1.848\right)\; \end{array}$$

When an oviposition grid is used, the estimated regression equation is as follows:

(5) $$\begin{array}{c} number\;of\;eggs\;per\;beetle\;per\;day=\\ 12.572\\ -0.882\times beetle\;density\;\left(number/cm²\right)\\ -0.162\times oviposition\;duration\;\left(days\right)\\ -2\times \ln\left(mean\;age\;of\;beetles\;\left(days\right)-10.5\right)\\ +0.695\;x\;beetle\;density\;\left(number/cm^{2}\right)\\ \times \left(\ln\left(mean\;age\;of\;beetles\;\left(days\right)-10.5\right)-1.848\right)\\ -0.817\times \left(ln\left(mean\;age\;of\;beetles\;\left(days\right)-10.5\right)-1.848\right)\; \end{array}$$

The overall regression was statistically significant (F(6,45) = 94.0494, *p* < 0.0001, R^2^ = 0.93). The number of eggs per beetle per day significantly correlates to presence of grid, oviposition duration and beetle age (Table 5).

**Figure 4:**
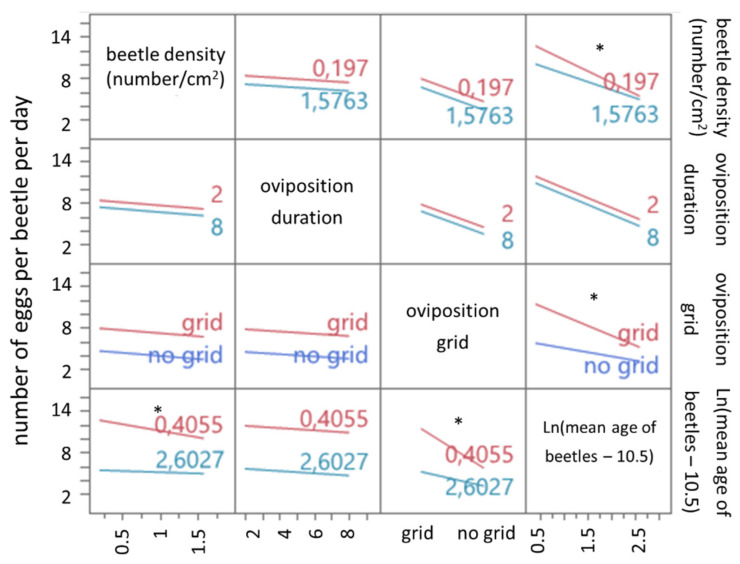
Interaction plots of the linear regression model for the number of eggs per beetle per day of experiment 2. Each interaction plot shows the interaction of the row effect with the column effect. The horizontal axis is scaled for the variable displayed in each column. Lines represent the number of eggs per beetle per day in function of the row variable, for high (blue line) or low (red line) values of the column variable. Values of the column variables are also indicated. Parallel lines indicate the absence of an interaction, while diverging lines indicate the presence of a (non)-significant interaction. Significant interactions (*p* < 0.05) are marked by an asterisk (*).

Similar to experiment 1, the presence of a grid has a positive influence on the number of eggs per beetle per day. Oviposition duration and beetle age also have a negative influence on the number of eggs per beetle per day. Beetle density has a negative influence on the number of eggs per beetle per day as well. A negative interaction between beetle age and beetle density is present. Beetle age also negatively interacts with the presence of a grid. The older the beetles are, the smaller the influence of beetle density and the presence of a grid becomes (Figure 4).

For the hatching of experiment 2, a significant regression model (Table 6) was found (F(2,49) = 7.8104, *p* = 0.0011, R^2^ = 0.24). Beetle age has a negative linear correlation (*p* = 0.0392). In contrast to experiment 1, the use of an oviposition grid also has a significant correlation (*p* = 0.0023). When an oviposition grid is used, the intercept of the model increases (Table 6). 

For the mean hatching time of experiment 2, a significant regression model (Table 7) was found (F(4,47) = 36.209, *p* < 0.0001, R^2^ = 0.75). For mean hatching time, a significant correlation is present for oviposition duration (*p* < 0.0001), beetle age (*p* < 0.0001) and presence of an oviposition grid (*p* = 0.0320) (Table 7). 

## 4. Discussions

Despite the increased interest in mealworm farming, the optimization of the mealworm reproduction process has not been widely explored. Reproductive success has mostly been assessed by determining the number of offspring several weeks after oviposition. As a result, the egg yield and their hatch rates remain unknown. Therefore, the objective of this study was to determine the effect of different reproduction factors on the number of eggs produced by *T. molitor* (per beetle per day) and the egg hatching (rate and time). Studied reproduction factors included (1) use of an oviposition grid, (2) oviposition duration, (3) beetle age and (4) beetle density. Additionally, the effect of the harvesting method (manual harvesting versus using a 0.500 µm vibration screen) on the hatching rate of *T. molitor* eggs was examined.

### 4.1. The Effect of Different Reproduction Factors on the Number of Eggs Produced by Tenebrio Molitor

All factors investigated in this study have an effect on the number of eggs per beetle per day. The use of a grid during oviposition significantly increases the number of eggs produced per beetle per day. *T. molitor* is known to behave cannibalistic towards its eggs [13,18,19]. Using a grid during reproduction may inhibit the adult beetles from damaging their eggs through movement, interference and cannibalism, as they are physically separated from the eggs laid in the wheat flour. As a result, a higher egg yield is established, as confirmed by this study and by Deruytter et al. [19].

Based on our results, the number of eggs produced per beetle per day decreased as oviposition duration increased. Even though their data are presented as larval yield, similar results were reported by Berggreen et al. [18] and Deruytter et al. [19]. Both studies indicated that the yield/beetle/day is significantly affected by oviposition duration, and the highest reproduction per beetle/day was obtained at shorter reproduction periods. This is confirmed by our study.

However, it must be considered that the oviposition duration is affected by beetle age as the beetles get older during the duration of the experiment. As reported by Berggreen et al. [18] and Morales-Ramos et al. [13], the age of the beetles has a negative effect on the reproduction of *T. molitor*. Both studies reported a decreased number of larvae as beetles grew older. The negative effect of age on the reproduction of *T. molitor* is confirmed by our study. Based on the results, it is clear that the age of the beetles has a profound impact on the egg yield per beetle per day. The negative correlation between beetle age and egg yield seems to be more profound when a grid is used during oviposition. However, the positive effect of the oviposition grid decreased over time.

It is known that reproductive output is age-dependent [13]; however, it must be noted that the decrease already starts with relatively young beetles. For instance, in our experiment, a mix of beetles of maximum 2 weeks old was used, and the experiment lasted 16 days. Within this time frame, reproduction already declined. These results are in line with those reported by Morales-Ramos et al. [13], which found reduced fecundity of beetles already starts after 3 weeks of age.

The influence of *T. molitor* beetle density has been investigated in several studies [13,18,19,20]. Results in this study show that the number of eggs produced per beetle per day was negatively affected by beetle density. This is also reported by several other studies where a decrease in reproduction was found when increasing the beetle density [13,18,19,20].

The negative correlation between beetle density and egg yield per beetle per day is most likely due to higher cannibalism and damaging of the eggs with higher densities. This was reported in the study of Deruytter et al. [19] for larval yield/beetle/day and confirmed by our results for the number of eggs produced/beetle/day. The effect of density is not significant when beetles are not able to interfere with their eggs, i.e., when using a grid during oviposition.

The linear regression model shows an interaction effect between beetle density and beetle age. The effect of beetle density on the number of eggs/beetle/day decreases as the beetle age increases, probably due to the reduced reproduction as beetles grow older, as discussed above.

Estimated by the model, a maximum yield of 13 eggs/beetle/day can be achieved by using (1) a grid during oviposition, (2) applying a beetle density of 0.2 beetles/cm², (3) using an oviposition duration of 2 days with a mean beetle age of 12 days.

### 4.2. The Effect of Different Reproduction Factors on the Hatching of Tenebrio Molitor Eggs

The hatching rate was not influenced by oviposition duration, but time till hatching was. The results of this research showed a negative correlation between oviposition duration and hatching time. This was expected as time till hatching logically increases when beetles are allowed to lay eggs for a shorter period of time. An increase in standard deviation as oviposition duration increases was also expected: offspring from beetles allowed to lay eggs for a longer period will have a larger spread in age. However, we expected a larger standard deviation than shown by the results (Figure S10). For example, eggs that are laid on the last 2 days of the oviposition duration of 8 days are expected to hatch within the same timespan as eggs laid by beetles of the oviposition duration of 2 days. This was not shown by the results. One explanation could be that fewer ′younger′ eggs were sampled. This might be due to the effect of cannibalism or beetle age or to the sampling method.

Beetle age has a negative effect on hatching rate and mean hatching time. A decline in hatching rate over time was also detected in the study of Adamaki-Sotiraki et al. [21].

In addition, the effect of the egg harvesting method on the egg hatching was also included in this study. Only a significant difference between manual harvesting and harvesting using a 500 µm vibrating sieve was observed when eggs were harvested from the no grid treatment. When a grid is used during oviposition, the harvesting method did not have a significant effect on egg hatching rate. A possible explanation for the significant difference might be that during the manual harvesting of eggs, damaged eggs (by the beetles as no grid was used) were also selected for further investigation.

## 5. Conclusions

Based on the results obtained in this study, it can be concluded that all investigated factors have an effect on the number of *T. molitor* eggs produced per beetle per day. Beetle age, oviposition duration and beetle density have a negative impact on yield. The use of an oviposition grid allows an increase in yield. The positive effect of this grid decreases with higher densities, suggesting that egg cannibalism or other mechanical damage cause a decreased yield at higher densities. The effect of the grid also decreases when beetle age increases, indicating that age itself causes a decline in egg production.

Hatching rate and time till hatching was influenced by the use of a grid and beetle age.

## Figures and Tables

**Table 1:** Experimental design. Two experiments were performed. Experiment 1: 6 batches of beetles were set up with a density of 0.1 g beetles/cm² of which 3 batches with an oviposition grid and 3 batches without an oviposition grid. Experiment 2: beetles were placed in crates at three densities, i.e., 0.025 g (equal to 0.197 beetles/cm²), 0.1 g beetles/cm² (equal to 0.788 beetles/cm^2^) and 0.2 g beetles/cm² (equal to 1.576 beetles/cm²). For each density, 6 beetle batches were set up: 3 batches with an oviposition grid and 3 batches without an oviposition grid. For both experiments, each beetle batch was allowed to lay eggs for 2, 4, and 8 days (oviposition duration is indicated in red, green and yellow, respectively). After each oviposition period (2, 4 or 8 days), beetles were removed from the crates and transferred to a new crate with a different oviposition duration. The order of the oviposition duration differed between the batches, allowing the effect of age (mean beetle age during each treatment is indicated in the treatment boxes) to be included in the study.

Experiment		Experiment 1									Experiment 2														
Code Beetle Batch		200M.A	200M.B	200M.C	200Z.A	200Z.B	200Z.C	50AM	200AM	400AM	50AZ	200AZ	400AZ	50BM	200BM	400BM	50BZ	200BZ	400BZ	50CM	200CM	400CM	50CZ	200CZ	400CZ
Grid/No Grid		Grid	Grid	Grid	No Grid	No Grid	No Grid	Grid	Grid	Grid	No Grid	No Grid	No Grid	Grid	Grid	Grid	No Grid	No Grid	No Grid	Grid	Grid	Grid	No Grid	No Grid	No Grid
	Density (g/cm^2^)	0.1	0.1	0.1	0.1	0.1	0.1	0.025	0.1	0.2	0.025	0.1	0.2	0.025	0.1	0.2	0.025	0.1	0.2	0.025	0.1	0.2	0.025	0.1	0.2
Day																									
1		15			15			12	12	12				13	13	13				15	15	15			
2			12			12					12	12	12				13	13	13				15	15	15
3				13			13	17	17	17															
4			15			15					17	17	17												
5														16	16	16									
6																	16	16	16						
7				19			19							21	21	21									
8			21			21											21	21	21						
9		20			20															21	21	21			
10																							21	21	21
11		23			23			23	23	23															
12											23	23	23												
13																				24	24	24			
14																							24	24	24
15				25			25																		
16																									

**Table 2:** Parameter estimates, standard error and significance level (t ratio and *p*-value) for multiple linear regression model of the number of eggs per beetle per day for experiment 1.

Term	Estimate	Std Error	t Ratio	Prob > |t|
Intercept	8.384	0.478	17.5	<0.0001
oviposition duration (days)	−0.19	0.064	−3.00	0.0096
oviposition grid[grid]	1.087	0.157	6.94	<0.0001
Ln(mean age of beetles−10.5)	−1.36	0.213	−6.39	<0.0001

**Table 3:** Parameter estimates, standard error and significance level (t ratio and *p*-value) for multiple linear regression model of hatching (%) for experiment 1.

Term	Estimate	Std Error	t Ratio	Prob > |t|
Intercept	0.887	0.031	28.68	<0.0001
mean beetle age (days)	0.004	0.002	2.67	0.0167

**Table 4:** Parameter estimates, standard error and significance level (t ratio and *p*-value) for a multiple linear regression model of mean hatching time (days) for experiment 1.

Term	Estimate	Std Error	t Ratio	Prob > |t|
Intercept	6.689	0.228	29.4	<0.0001
oviposition duration (days)	−0.318	0.043	−7.4	<0.0001

**Table 5:** Parameter estimates, standard error and significance level (t ratio and *p*-value) for multiple linear regression model of the number of eggs per beetle per day for experiment 2.

Term	Estimate	Std Error	t Ratio	Prob > |t|
Intercept	10.94	0.377	29.04	<0.0001
beetle density (number/cm^2^)	−0.882	0.181	−4.88	<0.0001
oviposition duration (days)	−0.162	0.042	−3.88	0.0003
oviposition grid[grid]	1.633	0.104	15.66	<0.0001
Ln(mean age of beetles − 10.5)	−2	0.153	−13.07	<0.0001
(beetle density (number/cm^2^) − 0.856) × (Ln(mean age of beetles − 10.5) − 1.848)	0.695	0.253	2.75	0.0085
Oviposition grid[grid] × (Ln(mean age of beetles − 10.5) − 1.84768)	−0.817	0.152	−5.37	<0.0001

**Table 6:** Parameter estimates, standard error and significance level (t ratio and *p*-value) for multiple linear regression model of hatching (%) for experiment 2.

Term	Estimate	Std Error	t Ratio	Prob > |t|
Intercept	0.99	0.035	28.09	<0.0001
oviposition grid[grid]	0.025	0.008	3.22	0.0023
mean age of beetles	−0.004	0.002	−2.12	0.0392

**Table 7:** Parameter estimates, standard error and significance level (t ratio and *p*-value) for linear regression model of mean hatching time (days) for experiment 2.

Term	Estimate	Std Error	t Ratio	Prob > |t|
Intercept	7.745	0.351	22.05	<0.0001
oviposition duration (days)	−0.279	0.024	−11.48	<0.0001
oviposition grid[grid]	−0.133	0.06	−2.21	0.0320
mean age of beetles	−0.074	0.017	−4.30	<0.0001

## Data Availability

Original data are available from the author on request.

## Supplementary Materials

The following supporting information can be downloaded at: https://www.mdpi.com/article/10.3390/insects13070615/s1, Figure S1: Scatterplot for number of eggs per beetle per day vs. oviposition duration (days) for experiment 1 (a) and experiment 2 (b). Figure S2: Box plot of number of eggs per beetle per day vs. oviposition grid for experiment 1 (a) and experiment 2 (b). Figure S3: Scatterplot of number of eggs per beetle per day vs. mean beetle age (days) for experiment 1 (a) and experiment 2 (b). Figure S4: Scatterplot of number of eggs per beetle per day vs. mean beetle age (days) for experiment 1 (a) and experiment 2 (b). Figure S5: Scatterplot of number of eggs per beetle per day vs. beetle density for experiment 2. Figure S6: Scatterplot for hatching (%) vs. oviposition duration (days) for experiment 1 (a) and experiment 2 (b). Figure S7: Box plot of hatching (%) vs. oviposition grid for experiment 1 (a) and experiment 2 (b). Figure S8: Scatterplot of hatching (%) vs. mean beetle age (days) for experiment 1 (a) and experiment 2 (b). Figure S9: Scatterplot of hatching (%) vs. beetle density for experiment 2. Figure S10: Scatterplot for mean hatching time (days) vs. oviposition duration (days) for experiment 1 (a) and experiment 2 (b). Figure S11: Box plot of mean hatching time (days) vs. oviposition grid for experiment 1 (a) and experiment 2 (b). Figure S12: Scatterplot of mean hatching time (days) vs. mean beetle age (days) for experiment 1 (a) and experiment 2 (b). Figure S13: Scatterplot of mean hatching time (days) vs. beetle density for experiment 2.
