# Supplementary material for: Determining the Effect of Different Reproduction Factors on the Yield and Hatching of Tenebrio Molitor Eggs
Source: Insects. 2022 Jul 8;13(7):615. doi: 10.3390/insects13070615 (PMC9322254; doi:10.3390/insects13070615)
Supplement: Supplementary file 1 [file insects-13-00615-s001.zip › insects-1736011-supplementary.pdf]

Supplementary Materials

Article

Determining the effect of different reproduction factors on the yield and hatching of *Tenebrio molitor* eggs

Lotte Frooninckx[1*], Siebe Berrens[1], Meggie Van Peer[1], Ann Wuyts[1], Laurens Broeckx[1] and Sabine Van Miert[1]

1. Supplementary results

Before performing the multiple linear regression each independent variable was examined individually against the dependent variables to see if a correlation could be observed.

1.1. Number of eggs per beetle per day

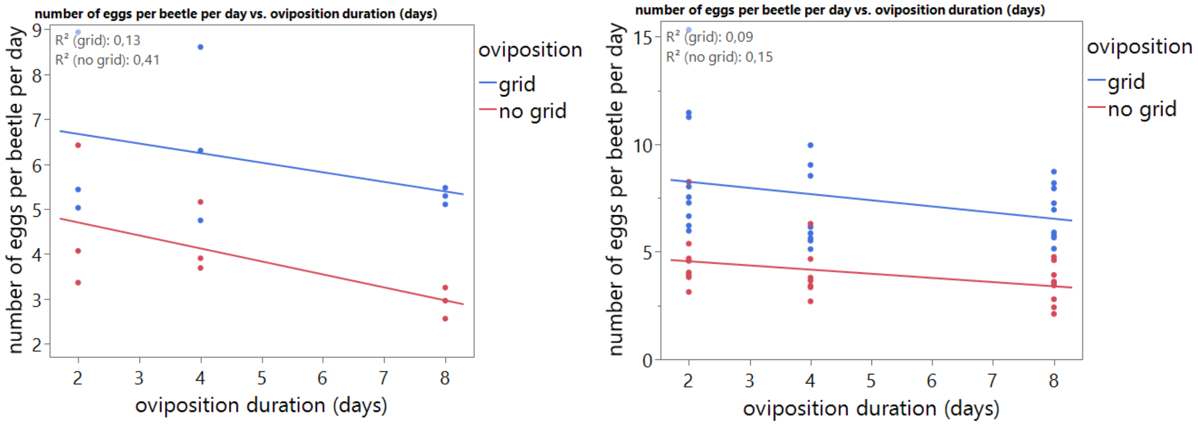

(**a**) (**b**)

Figure S1. Scatterplot for number of eggs per beetle per day vs. oviposition duration (days) for experiment 1 **(a)** and experiment 2 **(b)**. Points represent individual measurements. Use of an egg laying grid is indicated in blue (grid) and red (no grid). Solid line represents linear fit. RSquare is indicated in the top left.

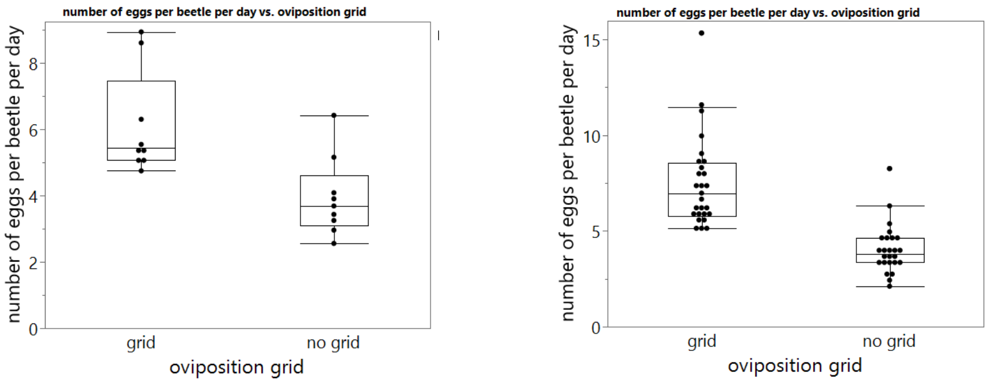

(**a**) (**b**)

Figure S2. Box plot of number of eggs per beetle per day vs. oviposition grid for experiment 1 (a) and experiment 2 (b). Points represent individual measurements.

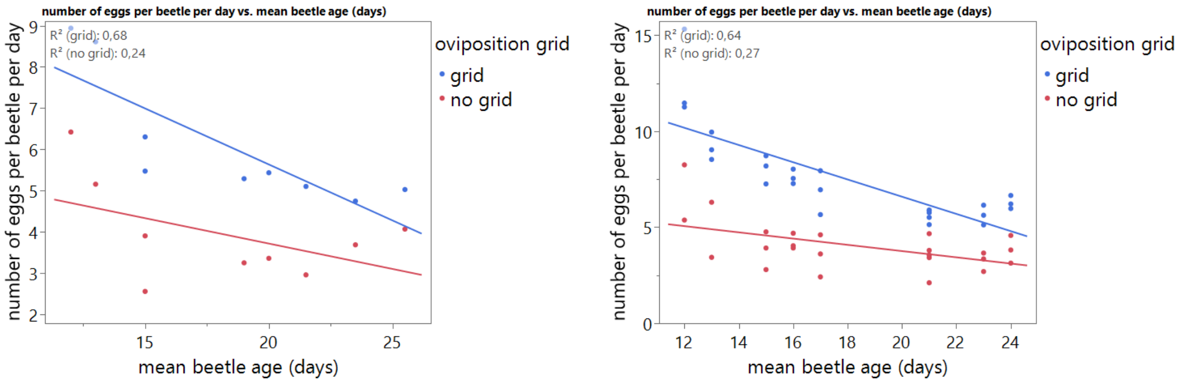

(**a**) (**b**)

Figure S3. Scatterplot of number of eggs per beetle per day vs. mean beetle age (days) for experiment 1 (a) and experiment 2 (b). Points represent individual measurements. Use of an egg laying grid is indicated in blue (grid) and red (no grid). Solid line represents linear fit. RSquare is indicated in the top left.

For the effect of beetle age on egg laying per beetle an asymptotic regression was also fitted (Y = M – (M – c) * exp(-kt)) (Figure S4).

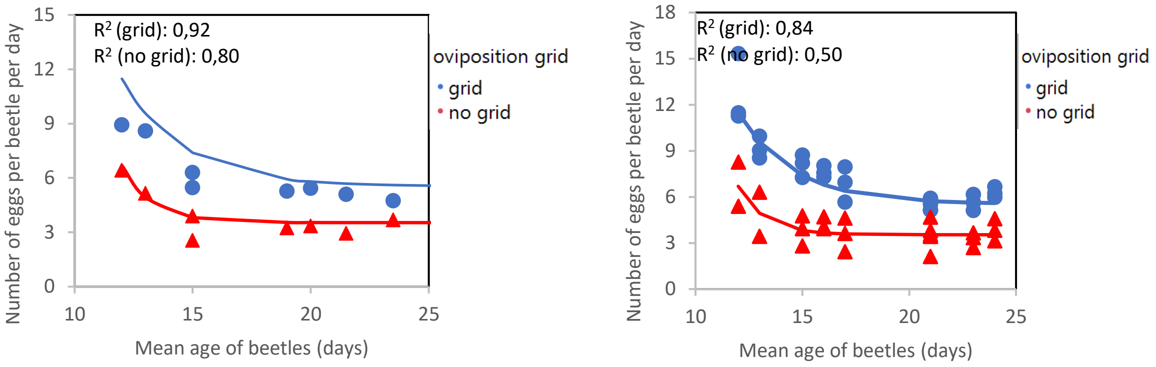

(**a**) (**b**)

Figure S4. Scatterplot of number of eggs per beetle per day vs. mean beetle age (days) for experiment 1 (a) and experiment 2 (b). Points represent individual measurements. Use of an egg laying grid is indicated in blue (grid) and red (no grid). Solid line represents asymptotic fit. RSquare is indicated in the top left.

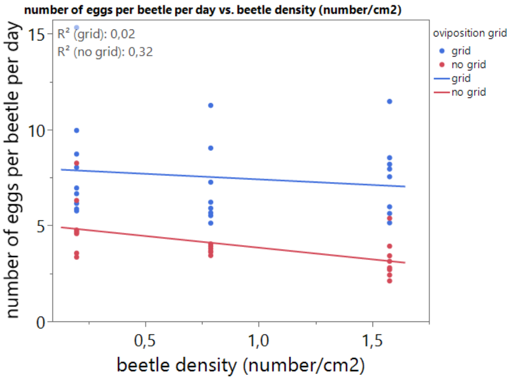

Figure S5. Scatterplot of number of eggs per beetle per day vs. beetle density for experiment 2. Points represent individual measurements. Use of an egg laying grid is indicated in blue (grid) and red (no grid). Solid line represents linear fit. RSquare is indicated in the top left.

1.2. Hatching

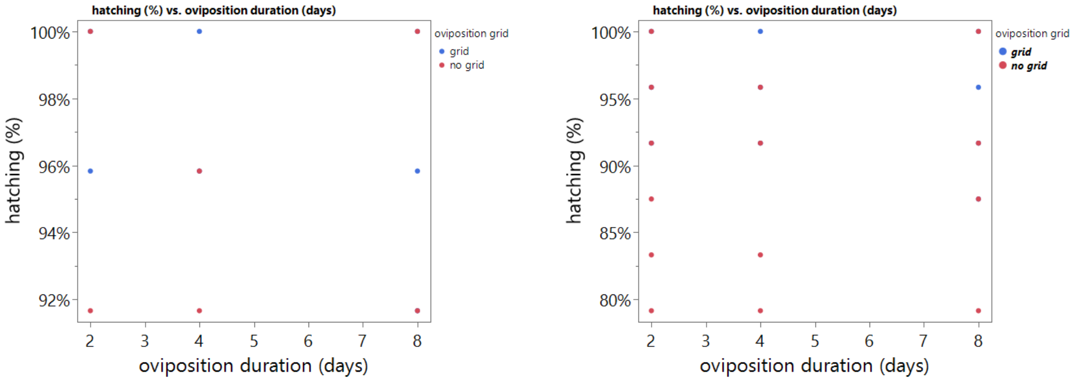

(**a**) (**b**)

Figure S6. Scatterplot for hatching (%) vs. oviposition duration (days) for experiment 1 **(a)** and experiment 2 **(b)**. Points represent individual measurements. Use of an egg laying grid is indicated in blue (grid) and red (no grid).

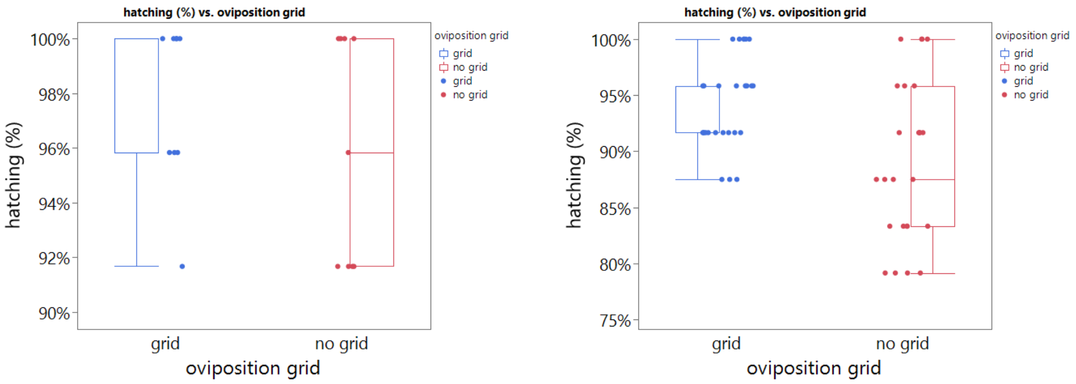

(**a**) (**b**)

Figure S7. Box plot of hatching (%) vs. oviposition grid for experiment 1 (a) and experiment 2 (b). Points represent individual measurements.

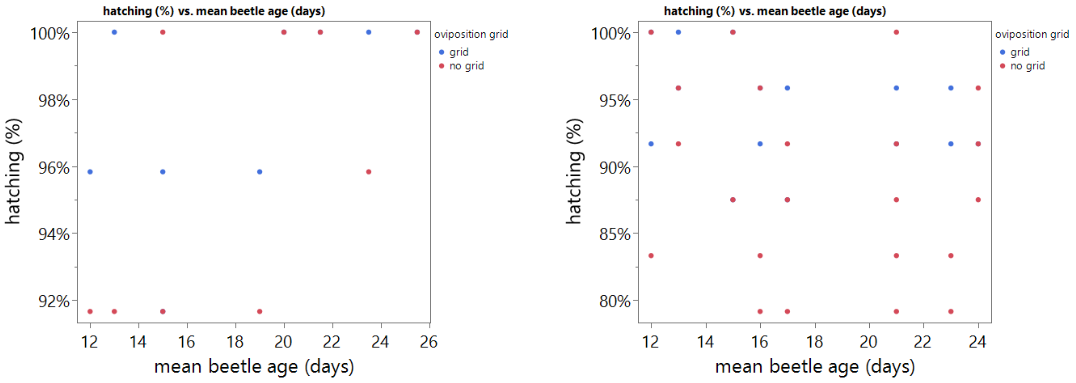

(**a**) (**b**)

Figure S8. Scatterplot of hatching (%) vs. mean beetle age (days) for experiment 1 (a) and experiment 2 (b). Points represent individual measurements. Use of an egg laying grid is indicated in blue (grid) and red (no grid).

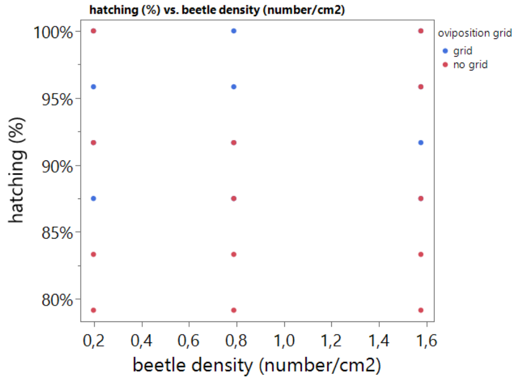

Figure S9. Scatterplot of hatching (%) vs. beetle density for experiment 2. Points represent individual measurements. Use of an egg laying grid is indicated in blue (grid) and red (no grid).

1.3. Mean hatching time

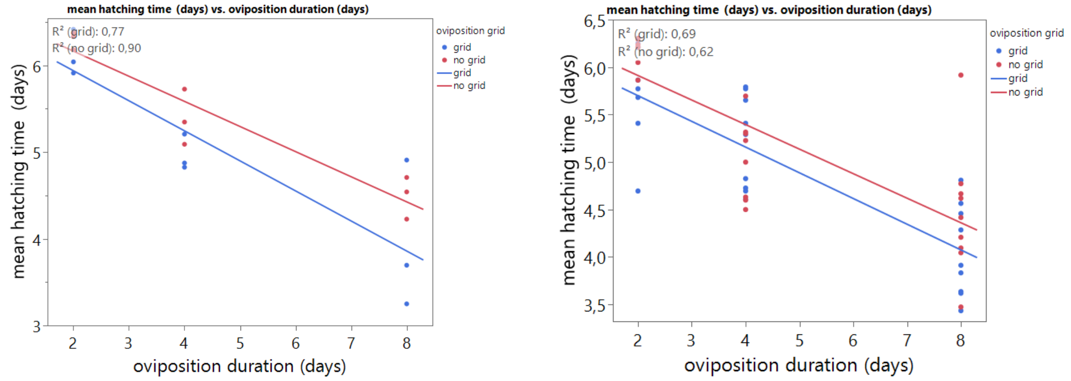

(**a**) (**b**)

Figure S10. Scatterplot for mean hatching time (days) vs. oviposition duration (days) for experiment 1 **(a)** and experiment 2 **(b)**. Points represent individual measurements. Use of an egg laying grid is indicated in blue (grid) and red (no grid). Use of an egg laying grid is indicated in blue (grid) and red (no grid). Solid line represents linear fit. RSquare is indicated in the top left.

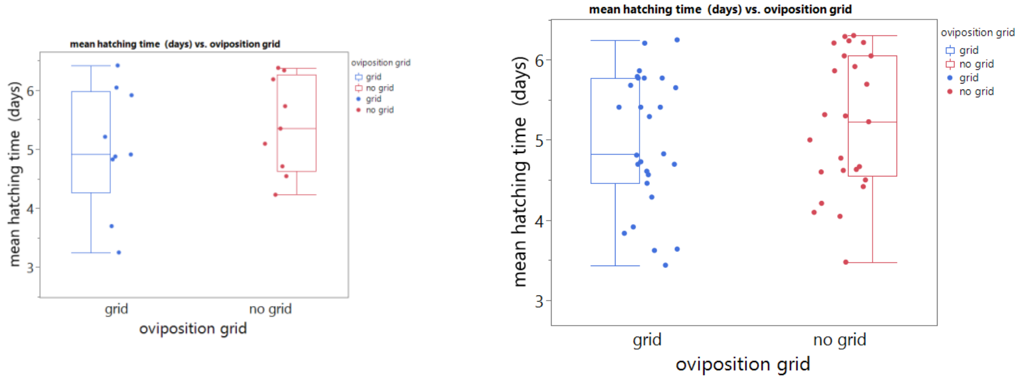

(**a**) (**b**)

Figure S11. Box plot of mean hatching time (days) vs. oviposition grid for experiment 1 (a) and experiment 2 (b). Points represent individual measurements.

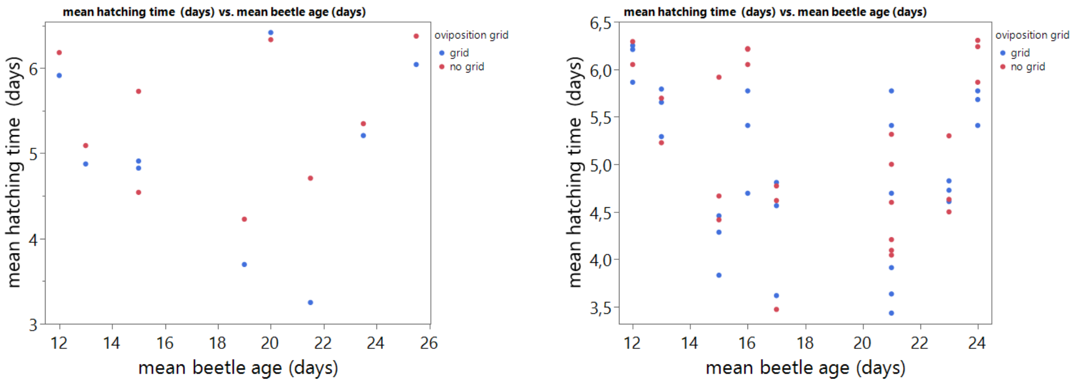

(**a**) (**b**)

Figure S12. Scatterplot of mean hatching time (days) vs. mean beetle age (days) for experiment 1 (a) and experiment 2 (b). Points represent individual measurements. Use of an egg laying grid is indicated in blue (grid) and red (no grid).

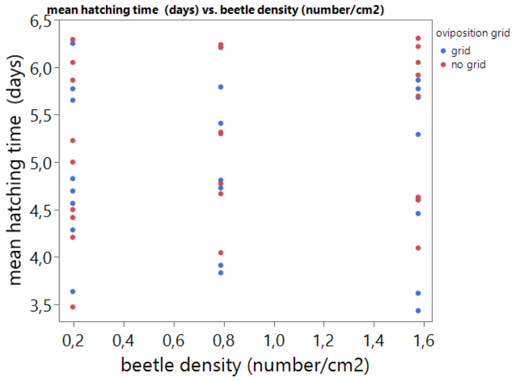

Figure S13. Scatterplot of mean hatching time (days) vs. beetle density for experiment 2. Points represent individual measurements. Use of an egg laying grid is indicated in blue (grid) and red (no grid).
